# Purification and pharmacokinetic study of gadoxetate isomers for enhanced rabbit liver MR imaging

**DOI:** 10.1371/journal.pone.0343927

**Published:** 2026-03-05

**Authors:** Yiling Chang, Qilin Li, Man Yi, JiGen Li, Xiaodong Yuan

**Affiliations:** 1 Guangzhou Special Service Recuperation Center of PLA Rocket Force, Guangzhou, China; 2 309th Hospital of PLA: 8th Medical Center of Chinese PLA General Hospital, Beijing, China; University of New Hampshire, UNITED STATES OF AMERICA

## Abstract

This study aimed to purify and prepare two isomers of Gadoxetate (Gd-A and Gd-B) from Pumexian and evaluate their stability, magnetic resonance (MR) imaging characteristics, and pharmacokinetics in rabbits. Reversed-phase liquid chromatography was employed to separate Gd-A and Gd-B, achieving purities exceeding 99% for both isomers. These purified isomers were then processed into vacuum-encapsulated solid powders via nitrogen blowing and freeze-drying. Stability assessments were conducted by storing the powders at various temperatures (25°C, −20°C, 50°C, and 80°C) for two months. The results indicated high stability at room temperature (25°C) and low temperature (−20°C), with both Gd-A and Gd-B maintaining 99% purity. At higher temperatures, purity slightly decreased: Gd-A was 98% at 50°C and 95% at 80°C, while Gd-B was 97% at 50°C and 94% at 80°C. For the in vivo evaluation, twelve rabbits were randomly assigned to two groups and received intravenous bolus injections of either pure Gd-A or Gd-B, followed by enhanced MR scanning and serial blood sampling. Pharmacokinetic and imaging analyses revealed statistically significant differences (p < 0.05) between the two isomers. Specifically, Gd-A exhibited a shorter time to peak enhancement in liver parenchyma (t_peak_A < t_peak_B), higher peak signal intensity in the liver parenchyma (SI_peak_A > SI_peak_B), greater plasma clearance (PCL-A > PCL-B), and a shorter half-life (t_1/2_A < t_1/2_B) compared to Gd-B. In conclusion, the vacuum-encapsulated solid powders of Gd-A and Gd-B demonstrate good long-term stability at room or low temperatures while maintaining high purity. Furthermore, the distinct pharmacokinetic profile and imaging characteristics of pure Gd-A suggest its potential value for clinical applications.

## Introduction

Gadoxetate disodium (Gd-EOB-DTPA) is a well-established liver-specific MRI contrast agent, valued for its unique dual pathway excretion involving both renal filtration and hepatocyte-specific uptake via organic anion transporting polypeptides (OATPs), followed by biliary excretion [1–7]. This mechanism provides distinct advantages for diagnosing and evaluating liver lesions, including hepatocellular carcinoma, liver metastases, and focal nodular hyperplasia, as well as assessing conditions like postoperative bile leakage [8–14]. Its amphiphilic nature, derived from the lipophilic ethoxybenzyl (EOB) group, facilitates this targeted liver uptake, distinguishing it from non-specific extracellular gadolinium-based contrast agents (GBCAs) [15–17].

However, a critical but often overlooked aspect of gadoxetate is its existence as a mixture of two diastereomers, Gd-A and Gd-B, typically present in a stable 65%:35% ratio under standard physiological conditions (pH 5–9, 25–120°C) [18]. Emerging evidence suggests these isomers possess distinct biological behaviors. Notably, the ratio of Gd-A to Gd-B found in bile significantly exceeds the administered ratio, implying that Gd-A undergoes more rapid hepatic uptake and clearance compared to Gd-B [19,20]. This observation raises a compelling hypothesis: could the purified Gd-A isomer offer superior performance as a contrast agent? Specifically, leveraging the potentially higher liver affinity and faster clearance of pure Gd-A might enable reduced contrast agent dosages, shorten imaging acquisition times, minimize systemic exposure, and consequently mitigate the risks associated with gadolinium retention and deposition [21], a growing concern with GBCAs, particularly linear agents like gadoxetate.

Despite this potential, the translation of pure Gd-A into clinical practice faces significant challenges. Although pioneering *in vitro* studies by Vander Elst et al. have revealed differences in albumin binding affinity between the Gd-A and Gd-B isomers, which theoretically account for their distinct pharmacokinetic behaviors, dedicated *in vivo* studies directly comparing their pharmacokinetics and imaging efficacy are still lacking [22]. Consequently, previous understanding of their individual performances has largely been inferred from the behavior of the racemic mixture [18]. In addition, pure Gd-A still faces practical obstacles when used as a liver-specific imaging contrast agent in humans. In a solution state, Gd-A undergoes slow conversion to Gd-B: while the metabolic process in the human body is relatively short, this conversion is negligible. However, if Gd-A is stored in solution for a long time, it will turn into a racemic mixture of Gd-A and Gd-B, thus losing its clinical value. Therefore, it is essential to explore long-term storage solutions for Gd-A. In this study, Gd-A was prepared into solid crystalline powder to achieve long-term stable storage of the single isomer (Gd-A), and no relevant studies have verified the feasibility of this solution before. To address these gaps, it is crucial to develop more efficient and safer liver-specific magnetic resonance imaging (MRI) contrast agents—especially by verifying the differences in pharmacokinetics and liver imaging when pure Gd-A and Gd-B isomers are used alone as drugs in vivo, and solving the stability issue of long-term Gd-A storage.

**Fig 1:**
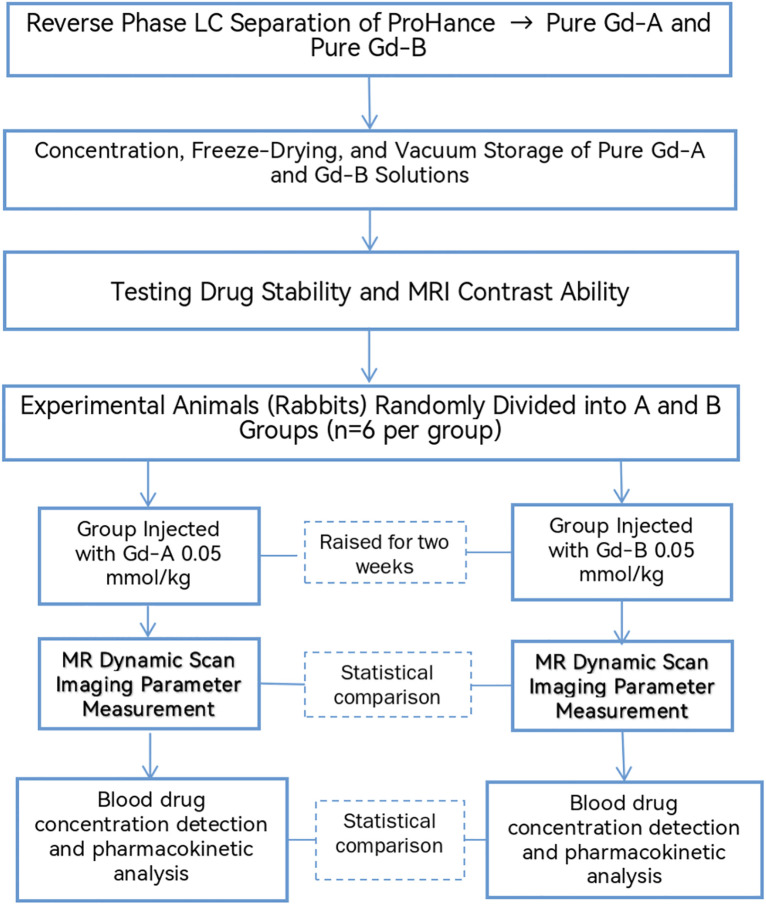
Overall Experimental Flowchart.

Therefore, this study introduces a novel approach to investigate the potential of purified Gd-A. We aimed to: 1) Develop and validate a method for separating Gd-A and Gd-B from gadoxetate and, critically, for maintaining the long-term purity of the isolated isomers in a stable form, addressing the previously unsolved challenge of interconversion. 2) Conduct the first direct comparative in vivo study in a rabbit model to evaluate the differences in MR imaging enhancement characteristics and pharmacokinetic profiles between purified Gd-A and Gd-B.By tackling both the purification/stabilization challenge and the lack of comparative in vivo data,this research seeks to provide foundational evidence for the potential clinical translation of pure Gd-A as an optimized liver-specific contrast agent (see Fig 1).

## Materials and methods

### Drug preparation and testing

#### Separation and purification.

**Fig 2:**
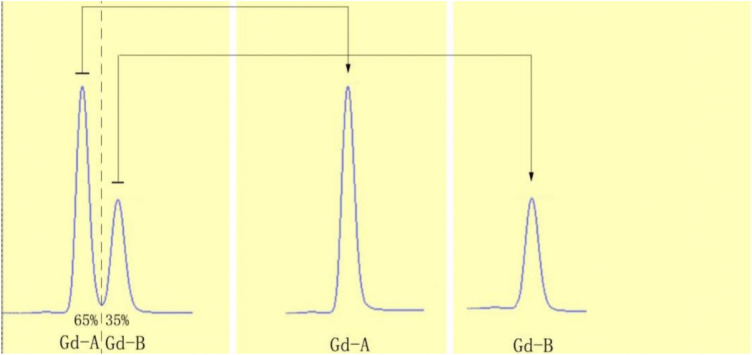
Reverse liquid chromatography analysis. The chromatogram on the left shows the original Primovist solution, containing two adjacent absorption peaks corresponding to the Gd-A and Gd-B isomers. The middle and right chromatograms display single, pure peaks for the isolated Gd-A and Gd-B isomers, respectively, confirming successful separation.

**Fig 3:**
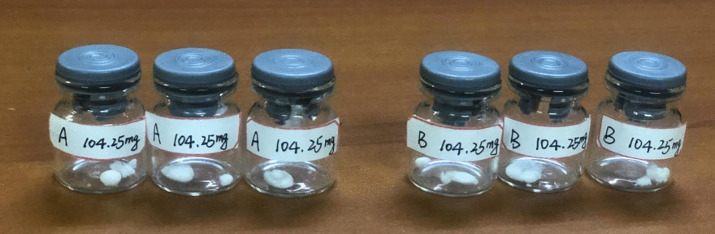
Solid powder of pure Gd-A and Gd-B. The concentrated pure Gd-A and Gd-B were converted into solid powder using a freeze-drying machine and sealed in glass vials for storage, with each vial containing approximately 104.25 mg.

This study employed reversed-phase high-performance liquid chromatography (HPLC) to purify the original solution of Primovist (with a concentration of 181.43 mg·ml^-1^). During the chromatographic process, dilute solutions of Gd-A and Gd-B were collected (see Fig 2). Utilizing a Shimadzu high-performance liquid chromatograph, we further confirmed that the purity of each batch of Gd-A and Gd-B exceeded 99%. Subsequently, these dilute solutions were placed in specially designed large glass containers and subjected to nitrogen blowing and concentration to 100 ml and 60 ml, respectively, to ensure purity. After filtration through a membrane to remove impurities, the concentrated solutions were stored in a freezing environment. Ultimately, the concentrated solutions were transferred into sterilized glass vials, and the white solid powders of Gd-A and Gd-B were obtained through freeze-drying under vacuum conditions, as depicted in Fig 3. The chromatographic conditions were as follows: the reversed-phase LC conditions were a mixture of methanol and ammonium bicarbonate buffer at a ratio of 15%:85%, with a flow rate of 1 ml/s, and the column chamber temperature was maintained at 25°C, with the detection wavelength set at 226 nm; the HPLC conditions were a mixture of methanol and phosphate buffer at a ratio of 25%:75%, with a flow rate of 0.5 ml/s, the column chamber temperature at 40°C, and the detection wavelength also at 226 nm. The chromatographic columns used were Allsphere ODS-2 (5μm, 250 mm × 22 mm) and Diamonsil C18 (5μm, 250 mm × 4.6 mm), with a guard column of Spursil C18 (5μm, 10 mm × 4.0 mm).

#### Stability and imaging capability.

After successfully concentrating the Gd-A and Gd-B solutions, we placed these alongside Primovist standard solutions (a series of solutions with varying concentrations prepared by diluting the original Primovist solution with ultra-pure water) in a water bath. Subsequently, we conducted abdominal MRI scans to verify whether the separated isomers retained the same imaging capabilities as the original Primovist solution.

We categorized and labeled the solid-state powders of these two isomers and stored them under four different dry environmental conditions: room temperature (25°C), low temperature (−20°C), and high temperatures (50°C and 80°C). All samples were vacuum-sealed in glass vials to ensure their stability over a period of more than two months. Subsequently, we used HPLC to detect changes in purity over time and conducted comparative analysis to evaluate the impact of different temperature conditions on the stability of the isomers.

### Animal experiments

The design of this experiment was reviewed and approved by the Ethics Committee of the Eighth Medical Center of the General Hospital of the People’s Liberation Army before its implementation. We selected 12 adult male New Zealand White rabbits, all of which were 6 months old, with body weights ranging from 2.5 to 2.75 kilograms. Prior to the commencement of the experiment, these rabbits underwent a two-week clinical observation to ensure their health status. All experimental animals were purchased from Xinglong Experimental Animal Farm in Haidian District, Beijing, which holds a valid experimental animal production license, number SCXK(Jing)2016−0003.

**Fig 4:**
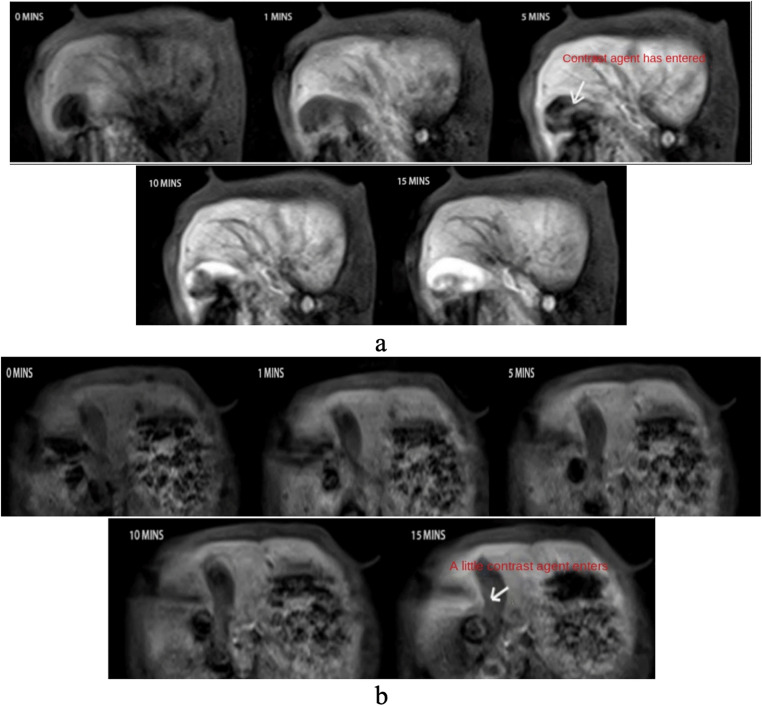
Dynamic MRI Imaging of Gd-A and Gd-B in the Rabbit Gallbladder. **a.** Following intravenous injection of Gd-A, the liver signal intensity significantly increased, with a small amount of contrast agent appearing in the gallbladder at 5 minutes (indicated by the arrow). **b.** Following intravenous injection of Gd-B, the liver signal intensity was relatively weaker, with a small amount of contrast agent appearing in the gallbladder at 15 minutes (indicated by the arrow).

Before the commencement of the experiment, each rabbit was fasted for 12 hours and then reweighed to accurately calculate the dosage of the anesthetic: 0.1 ml of a 2% solution of pentobarbital sodium per kilogram of body weight. All experiments were conducted using a Siemens (SIEMENS) Skyra 3.0T MR imaging device. A Y-shaped catheter was placed in the marginal vein of one ear of the rabbit, and anesthesia was induced using a 2% solution of pentobarbital sodium. After confirming that the rabbit was anesthetized, its limbs were securely fixed, and it was positioned supine on the scanning bed. A abdominal belt was used to control the rabbit’s breathing, followed by the application of an 8-channel abdominal coil specifically designed for human use, positioned over the liver area of the rabbit’s abdomen. A routine T1-weighted imaging (T1WI) scan was first performed to determine the location, and then a T1WI dynamic contrast-enhanced scanning sequence was initiated(with parameters set as follows:TR = 4.98ms,TE = 2.31ms,flip angle 15°, FOV = 189 mm × 220 mm, slice thickness 3.0 mm, slice gap 26 mm, phase encoding direction left-right). At the 2-minute mark of the scan, approximately 104250 μg doses of Gd-A and Gd-B were injected into each rabbit of groups A and B, respectively, through the other end of the catheter. After the injection, 2 ml of physiological saline was immediately injected into the catheter to flush out any residual contrast agent, with the injection process controlled within 8 seconds, followed by a continuous dynamic scan for an additional 15 minutes, as detailed in Fig 4. After administration, the drug concentration in the rabbit’s arterial blood was monitored dynamically, with four blood samples drawn at intervals between 20 and 70 minutes post-injection (at 20, 35, 50, and 70 minutes), each time drawing 0.5 to 1 ml of rabbit blood. The drawn blood samples were immediately placed in pre-labeled vacuum heparin lithium anticoagulant tubes to prevent coagulation. Considering the prolonged duration of the imaging examination, additional doses of 1–2 ml of anesthetic were administered to some animals as necessary. At the end of the experiment, all animals were humanely euthanized by intracardiac injection of air embolism or an overdose of anesthetic.

### Data processing and analysis

#### MRI image processing and analysis.

**Fig 5:**
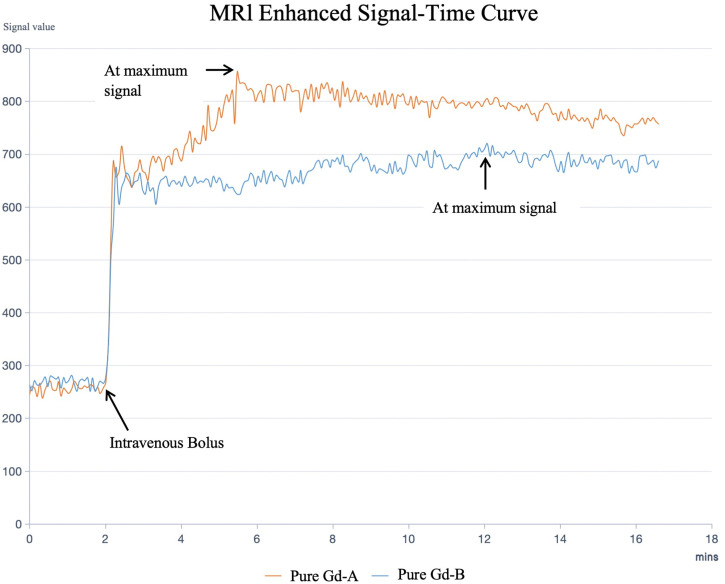
MRI Contrast-Enhanced Signal Time Curve. The liver MRI time-signal curves for Groups A and B were obtained by performing intravenous bolus injection at 2 minutes after the start of scanning, followed by continuous T1-weighted dynamic contrast-enhanced imaging without intervals.

The imaging data in this study were analyzed by a resident physician and an attending physician using a double-blind method to ensure the objectivity and accuracy of the results. During the evaluation, special attention was paid to avoiding bile ducts and blood vessels, and a clear display of the liver was selected for measuring the signal intensity (Signal Intensity, SI). To ensure the consistency of the measurements, the same Region of Interest (ROI) was placed on areas of the same size, and the data from plain and enhanced scans were measured respectively. The results were plotted with scan time as the horizontal axis and liver signal intensity as the vertical axis, and the signal intensity-time curves were generated using Excel software, as shown in Fig 5.

#### Blood sample processing and analysis.

**Fig 6:**
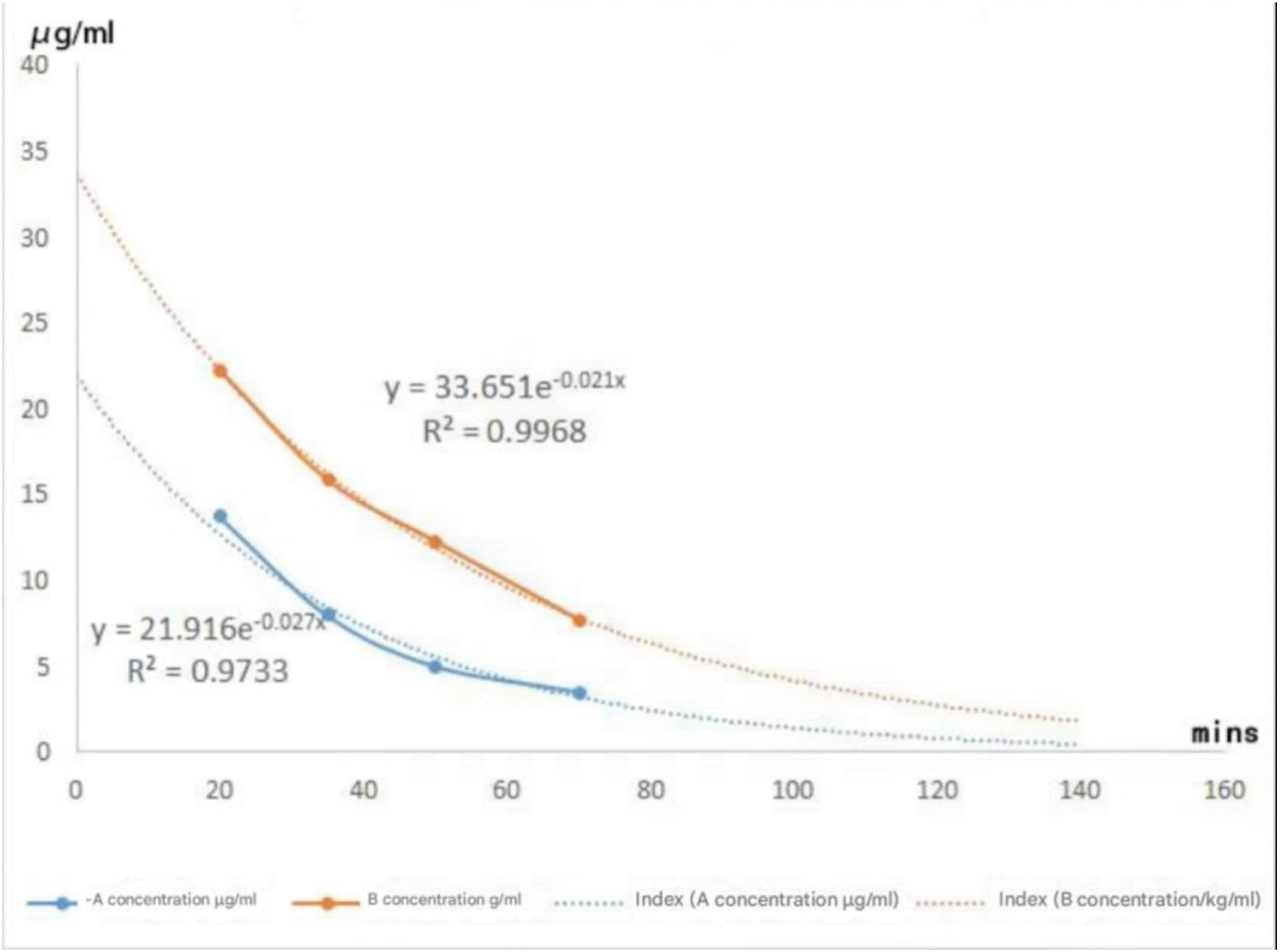
Exponential Decay Fitting of Blood Drug Concentration and Calculation of Area Under the Curve.

In the experiment, we first centrifuged the heparin tubes containing rabbit blood using a tabletop low-speed centrifuge at 4000 rpm for 7 minutes at room temperature (20–25°C) to separate the plasma. Then, 200μl of plasma samples were mixed with 600μl of methanol and thoroughly vortexed to achieve protein precipitation. Subsequently, the mixture was centrifuged using a high-speed refrigerated centrifuge at 14000 rpm for 2 minutes at room temperature (20–25°C) to obtain the supernatant, which was then transferred into sample vials and clearly labeled. HPLC was used to measure the drug concentrations in blood samples collected at different time points, with the detection conditions consistent with the previous experiments. Using Excel software, we plotted the blood drug concentration-time index curves for groups A and B, with the collection time of blood as the horizontal axis and the measured blood drug concentration as the vertical axis. Finally, the areas under the curves for groups A and B were calculated using the integral method, and the results are shown in Fig 6.

### Statistical analysis

In this study, we utilized the statistical software SPSS 18.0 for a comprehensive analysis of various indicators. Initially, the normality of the data was assessed using the Kolmogorov-Smirnov test, followed by the Levene test to evaluate the homogeneity of variances. For metric data that conformed to a normal distribution, we represented it as the mean (x) ± standard deviation (s). The differences in MR imaging indicators (including peak time t_peak_A, t_peak_B, and peak signal intensity SI_peak_A, SI_peak_B) and pharmacokinetic parameters (including the area under the concentration-time curve PCL-A, PCL-B, and half-life t_1/2_A, t_1/2_B) between Gd-A and Gd-B groups were compared using either the independent samples Student’s t-test or the non-parametric rank-sum test, depending on the normality of the data distribution. A p-value less than 0.05 was considered to indicate a statistically significant difference.

## Results

**Fig 7:**
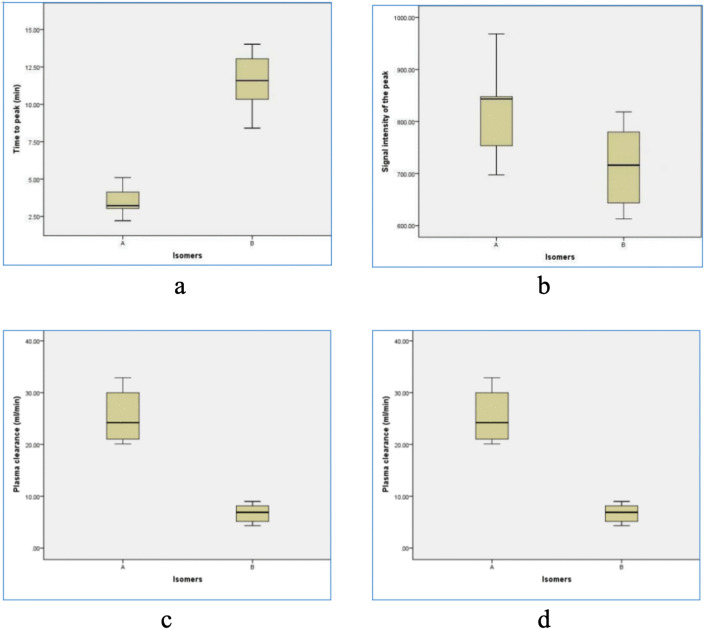
Comparison of the Distribution of Hepatic Pharmacokinetic Parameters. The figures include (in order) box plots of peak time in liver parenchyma, box plots of peak signal intensity in liver parenchyma, box plots of plasma clearance rate, and box plots of half-life.

Using reversed-phase HPLC technology, we successfully separated and obtained liquid solutions of Gd-A and Gd-B with purities exceeding 99%. These high-purity liquid solutions were transferred under vacuum conditions into glass vials and preserved in solid form at different temperatures for over two months. Under normal temperature (25°C), low temperature (−20°C), higher temperature (50°C), and high temperature (80°C) conditions, the purities of Gd-A were 99%, 99%, 98%, and 95%, respectively; the purities of Gd-B were 99%, 99%, 97%, and 94%, respectively, with specific data shown in Table 1. In MR imaging, the time for the liver parenchyma to reach peak value (t_peak_A) was significantly earlier than t_peak_B, at 3.49 ± 0.99 minutes versus 11.5 ± 2.03 minutes (p < 0.001). Similarly, the peak signal intensity of the liver parenchyma (SI_peak_A) was significantly higher than SI_peak_B, at 825.7 ± 92.9 versus 714.5 ± 78.8 (p = 0.049). In terms of pharmacokinetic parameters, the plasma clearance rate of Gd-A (PCL-A) was significantly higher than that of Gd-B, at 25.41 ± 5.174 ml/min versus 6.734 ± 1.834 ml/min (p < 0.001). Moreover, the half-life of Gd-A (t_1/2_A) was shorter than that of Gd-B, at 22.91 ± 3.42 minutes versus 35.27 ± 4.31 minutes (p < 0.001). All these results are statistically significant, with detailed information shown in Table 2 and Fig 7.

**Table 1:** Changes in Purity of Gd-A and Gd-B at Different Temperatures.

Isomer	−20°C	25°C	50°C	80°C
Gd-A	99%	99%	98%	95%
Gd-B	99%	99%	97%	94%

**Table 2:** MR Imaging and Pharmacokinetic Results.

MRI and pharmacokinetic indicators	Mean±SD	T_value_	p_value_
t_peakA_(min) t_peakB_(min)	3.49 ± 0.99 11.5 ± 2.03	8.679	p < 0.001
SI_peakA_ SI_peakB_	825.7 ± 92.9 714.5 ± 78.8	2.235	p = 0.049
PCL-A (ml/min) PCL-B(ml/min)	25.41 ± 5.174 6.734 ± 1.834	8.329	p < 0.001
t_1/2_A(min) t_1/2_B(min)	22.91 ± 3.42 35.27 ± 4.31	5.502	p < 0.001

## Discussion

The clinical utility of gadoxetate disodium (Gd-EOB-DTPA) in liver MRI is well-established, predicated on its unique hepatocyte-specific uptake mechanism. However, the inherent nature of gadoxetate as a mixture of two diastereomers, Gd-A and Gd-B, with differing pharmacokinetic profiles, has represented an underexplored avenue for optimizing liver contrast enhancement. A primary obstacle has been the lack of methods to isolate and, crucially, stabilize these isomers long-term to permit dedicated investigation [18]. This study directly addresses this long-standing challenge, presenting a significant advancement in the field. We report not only the successful separation of Gd-A and Gd-B to high purity (>99%) using reversed-phase HPLC but, more importantly, introduce a novel and effective stabilization strategy employing vacuum freeze-drying. This technique successfully preserved the purity of the isolated isomers in a solid, vacuum-sealed state for over two months across a range of temperatures (Table 1), effectively arresting the interconversion typically observed in solution. The development of this stabilization protocol is a key innovation of our work, as it overcomes a critical technical barrier and, for the first time, enables rigorous comparative in vivo evaluation of the individual isomers.

**Fig 8:**
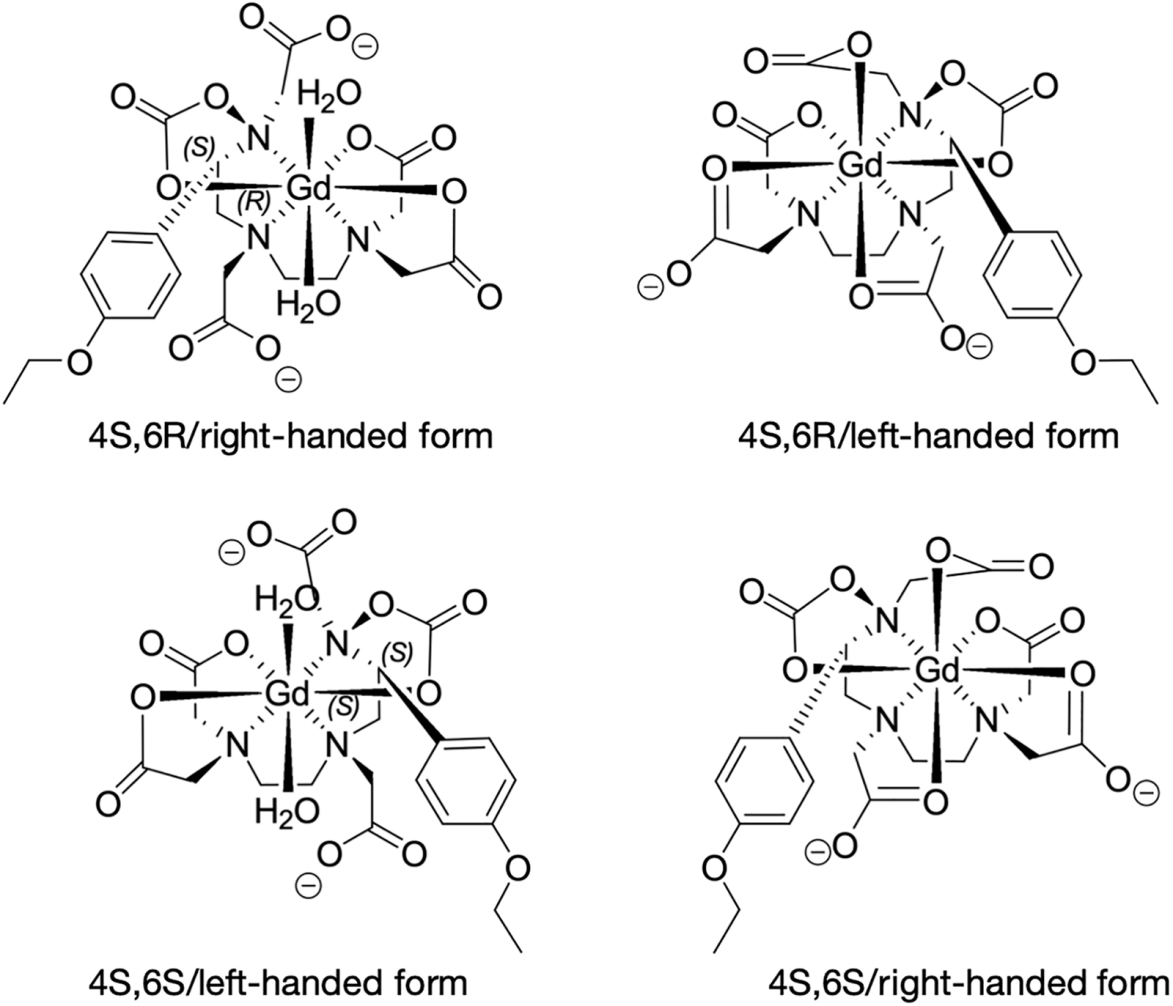
Sturctures of gadoxetate geometrical isomers. Sturctures of gadoxetate geometrical isomers in 4S,6R/right-handed, 4S,6R/left-handed, 4S,6S/left-handed, 4S,6S/right-handed forms.

A crucial aspect raised by this research is how the structural differences between the Gd-A and Gd-B isomers lead to their distinct in vivo behaviors (see Fig 8). Gd-EOB-DTPA possesses a single chiral center on the ethoxybenzyl (EOB) side chain, giving rise to two diastereomers, (R) and (S). This stereoisomerism, a variation in the three-dimensional arrangement of the molecule, is the fundamental driver of the differences in pharmacokinetics and MR imaging in liver. The specific spatial orientation of the EOB group in Gd-A likely facilitates its binding with the organic anion transporting polypeptides (OATPs) on the membrane of the hepatocyte. Our results, which show that Gd-A is taken up by the liver approximately three times faster than Gd-B (tpeak of 3.49 min vs. 11.5 min), strongly suggest that Gd-A possesses the optimal conformation for rapid and efficient OATP-mediated transport. This aligns with previous findings demonstrating isomer-specific transport systems for gadoxetate elimination [23].

Furthermore, the structure of these isomers influences their binding to plasma proteins, which in turn affects both their biodistribution and MR relaxivity in blood. Vander Elst et al. previously demonstrated that the two isomers exhibit stereospecific binding to human serum albumin (HSA), with one (Gd-A) having a higher affinity than the other (Gd-B) [22]. The isomer with higher protein binding would be expected to have a higher relaxivity in blood and a higher fraction available for immediate hepatic uptake. The rapid clearance and shorter half-life of Gd-A in vivo demonstrated in the current study are consistent with it having higher albumin affinity.

While Gd-A has a higher relaxivity in blood, its superior imaging performance in the current study—specifically its significantly higher peak signal intensity (SI_peak_) in the liver parenchyma—is best explained by its advantageous pharmacokinetic profile. The intrinsic relaxivities of the Gd-A and Gd-B molecules, once unbound from serum albumin when inside hepatocytes, are expected to be nearly identical. Therefore, the greater signal enhancement from Gd-A is not due to binding to plasma proteins but rather to its more rapid and efficient transport into the liver, leading to a higher intra-hepatocellular concentration. In essence, Gd-A’s structural configuration makes it a more effective substrate for the biological transport systems responsible for liver-specific uptake, validating its potential as a more efficient imaging agent.

As an intravenously administered agent, Gd-A and Gd-B are primarily distributed and diluted within the systemic circulation, with the physiological pH of blood (stably buffered between 7.35 and 7.45) representing the most relevant environment during the critical imaging window. While minor fluctuations in pH and salinity may occur in the microenvironments of organ capillaries and interstitial spaces, the gadoxetate complex itself is known to be chemically stable across a broad pH range of 5–9 [18]. This range comfortably encompasses the physiological variations the agent would encounter. Therefore, it is reasonable to conclude that the stereoisomeric integrity of Gd-A and Gd-B is well-maintained *in vivo*. The significant differences in their pharmacokinetic and imaging behaviors observed in our study are thus attributable to their distinct stereospecific interactions with biological targets, such as OATP transporters, rather than to any instability within the physiological environment.

This methodological breakthrough lays the foundation for our core contribution: the direct empirical validation and extension of prior theoretical findings. While Yuan et al.’s earlier human study first proposed differential clearance rates between Gd-A and Gd-B, their conclusions were derived from pharmacokinetic calculations and theoretical extrapolations following the injection of an isomer mixture [20]. In contrast, our current animal study transcends theoretical speculation by administering highly purified isomers, providing the first empirical evidence of their distinct pharmacokinetic behaviors.We demonstrate that Gd-A exhibits significantly enhanced plasma clearance and a shorter half-life (Table 2, Fig 7), offering robust in vivo support for conclusions drawn from human data and confirming the inference that Gd-A is the more rapidly cleared isomer.

Crucially, our study expands significantly on the dimensions of the previous human research by integrating these pharmacokinetic findings with a direct evaluation of MR imaging characteristics—an aspect not assessed in the 2020 study. We provide the first direct in vivo comparison of the MR imaging characteristics of purified Gd-A versus Gd-B in a rabbit model. The findings compellingly demonstrate the superiority of the Gd-A isomer for liver imaging applications. Gd-A exhibited a significantly more rapid uptake into the liver parenchyma, evidenced by a time-to-peak enhancement (t_peak_) approximately three times faster than that of Gd-B (3.49 min vs. 11.5 min, p < 0.001) (Table 2, Fig 7). This rapid enhancement profile holds considerable clinical potential, suggesting that imaging protocols utilizing pure Gd-A could significantly shorten the acquisition window needed for the diagnostically vital hepatobiliary phase. This could translate to reduced overall scan times, improved patient comfort and tolerance, minimized potential for motion artifacts, and enhanced departmental workflow efficiency.

Furthermore, Gd-A achieved a significantly higher peak signal intensity (SI_peak_) in the liver parenchyma compared to Gd-B (825.7 vs. 714.5, p = 0.049) (Table 2, Fig 7). This superior enhancement efficacy implies that pure Gd-A might achieve the necessary diagnostic contrast at potentially lower administered doses compared to the standard 65:35 mixture of Gd-A/Gd-B currently in clinical use. Dose reduction is a critical goal in contrast agent development, directly impacting patient safety by lowering overall gadolinium exposure.

The pharmacokinetic analysis further reinforces the advantages of Gd-A, particularly concerning safety. Gd-A demonstrated markedly more efficient systemic elimination, characterized by a plasma clearance rate (PCL) nearly four times higher than that of Gd-B (25.41 ml/min vs. 6.734 ml/min, p < 0.001) and a substantially shorter plasma half-life (t_1/2_) (22.91 min vs. 35.27 min, p < 0.001) (Table 2, Fig 7). This rapid clearance profile is highly significant in the context of growing concerns about gadolinium deposition following GBCA administration, particularly with linear agents like gadoxetate [24,25]. While gadoxetate offers invaluable diagnostic information through its liver specificity, its linear structure raises concerns compared to more stable macrocyclic GBCAs. However, the dramatically reduced systemic residence time of pure Gd-A, as evidenced by its rapid clearance and short half-life, suggests a significantly lower window of opportunity for dechelation and subsequent gadolinium deposition in tissues like the brain or kidneys [26–30]. This intrinsic pharmacokinetic advantage, specifically its rapid elimination, may offer safety advantages for pure Gd-A compared with the conventional Gd-A/Gd-B mixture. By shortening systemic exposure time to the gadolinium complex, it raises the possibility of reduced long-term retention, which could be particularly relevant for patients requiring repeated MRI examinations. However, a comprehensive safety assessment，especially regarding nephrogenic systemic fibrosis (NSF)，must rigorously account for the thermodynamic and kinetic stability of the Gd-A complex (i.e., its dissociation and transmetallation rates), for which no data are currently available [31–34]. Although macrocyclic agents have been proven to possess stability advantages, none of them can be used for liver-specific imaging. If pure Gd-A can be demonstrated to have sufficient stability, it may eventually exhibit excellent properties that combine liver-specific diagnostic performance with rapid clearance kinetics, which would then hold extremely high clinical value. However, this still requires further verification through future clinical studies.The findings of this study also have significant implications for the commercial landscape of liver-specific contrast agents. The current clinical standard, Gd-EOB-DTPA (Primovist®/Eovist®), is the very mixture from which Gd-A and Gd-B were isolated. Our in vivo results provide the first direct evidence that purified Gd-A could be commercialized as a superior, “optimized” agent. The nearly threefold reduction in time to peak enhancement (3.49 min vs. 11.5 min) could translate into substantially shorter scan protocols, improving departmental workflow and patient throughput, which is a significant economic driver for imaging centers. Furthermore, the higher peak signal intensity achieved by Gd-A suggests the potential for dose reduction, a critical objective in radiology to enhance patient safety by minimizing gadolinium exposure. When compared to other commercially available agents with partial hepatobiliary excretion, such as gadobenate dimeglumine, Gd-EOB-DTPA is already favored for its high liver specificity (up to 50% biliary excretion). Our research identifies Gd-A as the key driver of this efficacy. By isolating this more potent isomer, we effectively remove the less efficient, slower-clearing Gd-B component. A commercial product based on pure Gd-A would therefore hold a unique and powerful market position, offering a demonstrably safer and more efficient profile than the current Gd-EOB-DTPA mixture and other competing agents.

Compared to standard extracellular GBCAs, pure Gd-A retains the critical advantage of hepatobiliary specificity mediated by OATP transporters, enabling functional assessment of hepatocytes and biliary excretion pathways essential for characterizing focal liver lesions and diffuse liver disease [35]. Our findings strongly suggest that Gd-A is the primary driver of this specific uptake and subsequent rapid excretion observed with Gd-EOB-DTPA. By isolating Gd-A, we effectively harness the more pharmacokinetically favorable isomer, removing the “drag” imposed by the slower-clearing Gd-B component present in the standard formulation. This purification could lead to not only improved imaging efficiency and safety but potentially a more sensitive tool for quantitative assessment of liver function compared to the current mixture.In essence, this study has expanded the research dimensions from “pharmacokinetic calculations and theoretical deduction under intravenous injection of isomer mixtures in human experiments” to a full-chain study of “preparation and stability testing of pure isomers—pharmacokinetic and MR imaging evaluation of pure isomers” in animal experiments.

Methodological note on plasma sample preparation. Because both diastereomers of Gd-EOB-DTPA bind to human serum albumin, with the (S) isomer showing higher affinity than the (R) isomer according to Vander Elst et al., protein precipitation with methanol likely removes a substantial bound fraction into the pellet [22]. Consequently, the analyte remaining in the supernatant represents an operationally defined, methanol-soluble (predominantly unbound) fraction, rather than the total plasma concentration. This introduces a potential under-estimation of absolute levels and a differential bias between isomers. Our Fig 6 profiles should therefore be interpreted as apparent concentration–time curves of the supernatant fraction, suitable for generating hypotheses on relative behavior under identical processing, but not as definitive total-PK metrics (e.g., for absolute clearance or AUC). In future work, total Gd will be quantified after complete acid digestion (e.g., HNO₃ microwave digestion) followed by ICP-OES/ICP-MS, and the free versus bound fractions will be established by equilibrium dialysis or ultrafiltration, ideally coupled with isomer-resolving LC-ICP-MS (Liquid Chromatography – Inductively Coupled Plasma – Mass Spectrometry)reconcile speciation with element-specific quantitation. These adjustments align with current bioanalytical best practices emphasizing validated recovery, matrix effects, and stability assessments.

While this study provides robust preclinical evidence and introduces a pivotal stabilization technique, certain limitations warrant discussion. The reversed-phase HPLC method used for purification, though effective at the laboratory scale, requires substantial optimization regarding scalability and cost-effectiveness for potential pharmaceutical manufacturing. Furthermore, while the rabbit model provides valuable in vivo data, interspecies differences in transporter expression, liver physiology, and overall pharmacokinetics necessitate caution when extrapolating these findings directly to humans. Additionally, this study focused on comparing pure Gd-A and Gd-B; a direct comparison between pure Gd-A and the standard clinical Gd-EOB-DTPA mixture under identical conditions would further solidify the demonstrated advantages. Future investigations should also explore the impact of varying doses of pure Gd-A.

Therefore, the crucial subsequent steps involve refining the purification and stabilization technology towards commercial feasibility and embarking on comprehensive human clinical trials. Phase I trials will be essential to confirm safety and establish human pharmacokinetics, followed by Phase II and III trials to determine optimal dosing, confirm diagnostic efficacy across various liver pathologies (e.g., HCC, metastases, FNH, adenoma subtypes) and patient populations (including those with cirrhosis or renal impairment), and directly compare performance against the current standard Gd-EOB-DTPA. Long-term safety monitoring will also be paramount.

In conclusion, this research marks a significant advancement by overcoming the long-standing challenge of stabilizing purified gadoxetate isomers and providing the first direct in vivo demonstration of Gd-A’s superior imaging and pharmacokinetic properties compared to Gd-B. The novel freeze-drying stabilization method enables future exploration and potential clinical translation. The faster and stronger liver enhancement, coupled with markedly rapid systemic clearance, strongly suggests that purified Gd-A holds the potential to become a next-generation liver-specific MRI contrast agent. It offers the prospect of reduced scan times, lower required doses, and, critically, an improved safety profile concerning gadolinium retention compared to the currently used Gd-EOB-DTPA mixture. These compelling findings provide a strong rationale for continued investigation and clinical development of pure Gd-A, potentially leading to a safer, more efficient, and more precise approach to MR imaging of the liver.

## Conclusion

In summary, Gd-A can be obtained through the separation and purification of Primovist, and it can be stored long-term and stably. Preliminary animal experiments have shown that Gd-A has advantages over Primovist in pharmacokinetics and MR imaging, indicating potential clinical application value. Future research should focus on improving the purity and efficiency of extraction techniques, as well as conducting more human studies to verify the safety and efficacy of Gd-A. Our research findings support Gd-A as a safer and more effective choice of gadolinium-based contrast agent, especially for patient populations who require frequent MRI examinations or are at risk of kidney disease. Future studies should continue to explore the application potential of Gd-A in various clinical settings and evaluate the safety and efficacy of its long-term use. Additionally, larger-scale clinical trials should be considered to confirm these preliminary findings and provide a scientific basis for updating clinical guidelines.

## Supporting information

S1 File ARRIVE Supporting Information. (DOCX)

S2 File HPLC date. (DOCX)

S3 File MRI-ROI date. (XLSX)

S4 File Blood Drug Concentration. (XLSX)
