# Supplementary material for: Purification and pharmacokinetic study of gadoxetate isomers for enhanced rabbit liver MR imaging
Source: PLoS One. 2026 Mar 5;21(3):e0343927. doi: 10.1371/journal.pone.0343927 (PMC12962483; doi:10.1371/journal.pone.0343927)
Supplement: S1 File — (DOCX) [file pone.0343927.s001.docx]

ARRIVE Supporting Information

**Study Title:** Purification and Pharmacokinetic Study of Gadoxetate Isomers for Enhanced Rabbit Liver MR Imaging

**1. Study design**

- Groups compared: Two groups of New Zealand White rabbits administered intravenous injections of purified Gd-A or Gd-B isomers.
- Control group: None; direct comparison between the two isomer groups.
- Experimental unit: Single animal (n=6/group).

**2. Sample size**

- Number per group: 6 rabbits in Gd-A group and 6 in Gd-B group (12 animals total).
- Justification: Estimated based on pilot data and statistical power from similar studies.

**3. Inclusion and exclusion criteria**

- Inclusion criteria: Healthy male New Zealand White rabbits (6 months old, 2.5–2.75 kg) from a licensed supplier (SCXK(Jing)2016-0003).
- Exclusion criteria: None (all data were analyzed).

n per group: 6 animals per group, all data retained.

**4. Randomisation**

- Allocation method: Random number table used to assign 12 rabbits to Gd-A (n=6) or Gd-B (n=6) groups.
- Confounder control: Experiments conducted during the same time period, consistent housing conditions, and standardized scanning parameters.

**5. Blinding**

- Data collection: Operators were aware of group assignments (due to different drug administrations).
- Outcome assessment: Imaging analysis performed by two physicians using a double-blind method, unaware of group assignments.

**6. Outcome measures**

- Liver parenchyma peak enhancement time (tpeak), peak signal intensity (SIpeak).
- Pharmacokinetic parameters: plasma clearance (PCL), half-life (t1/2).

**7. Statistical methods**

- Software: SPSS 18.0, Excel.
- Normality (Kolmogorov-Smirnov test) and homogeneity of variance (Levene test) assessed.
- Parametric data reported as mean±SD; independent samples t-test used for comparisons. Non-parametric data analyzed via rank-sum test. (*P*<0.05 considered statistically significant.)

**8. Experimental animals**

- Species/strain: New Zealand White rabbit (Oryctolagus cuniculus), male, 6 months old.
- Source: Xinglong Experimental Animal Farm, Haidian District, Beijing (SCXK(Jing)2016-0003).
- Health status: Clinically observed for 2 weeks prior to experiments with no abnormalities noted.

**9. Experimental procedures**

- Drug preparation:

Gd-A and Gd-B separated by reversed-phase HPLC (purity>99%), lyophilized into vacuum-sealed solid powders.

Stability tested at 25°C, -20°C, 50°C, and 80°C for 2 months; purity assessed by HPLC.

- Animal procedures:

Anesthesia: 2% pentobarbital sodium (0.1 ml/kg, ear vein injection).

Imaging: 3.0T MRI with dynamic contrast-enhanced T1WI (TR=4.98 ms, TE=2.31 ms, slice thickness 3.0 mm).

Administration: Bolus injection of Gd-A or Gd-B (104,250 μg) followed by saline flush.

Blood sampling: At 20, 35, 50, and 70 min post-injection; plasma separated and analyzed by HPLC.

**10. Results**

- Descriptive statistics:

tpeak: Gd-A 3.49±0.99 min vs. Gd-B 11.5±2.03 min (*P*<0.001).

SIpeak: Gd-A 825.7±92.9 vs. Gd-B 714.5±78.8 (*P*=0.049).

PCL: Gd-A 25.41±5.174 ml/min vs. Gd-B 6.734±1.834 ml/min (*P*<0.001).

t1/2: Gd-A 22.91±3.42 min vs. Gd-B 35.27±4.31 min (*P*<0.001).

- Effect sizes: Mean differences with 95% confidence intervals provided (see Table 2 in the paper).
