# Supplementary material for: Purification and pharmacokinetic study of gadoxetate isomers for enhanced rabbit liver MR imaging
Source: PLoS One. 2026 Mar 5;21(3):e0343927. doi: 10.1371/journal.pone.0343927 (PMC12962483; doi:10.1371/journal.pone.0343927)
Supplement: S2 File — (DOCX) [file pone.0343927.s002.docx]

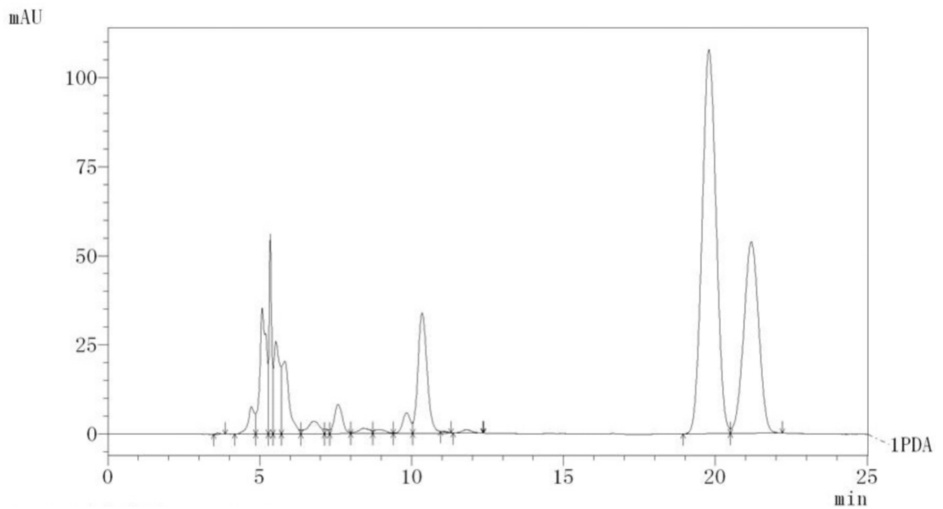


Figure 1. Blood sample after Primovist injection (PDA / 226nm 4nm).


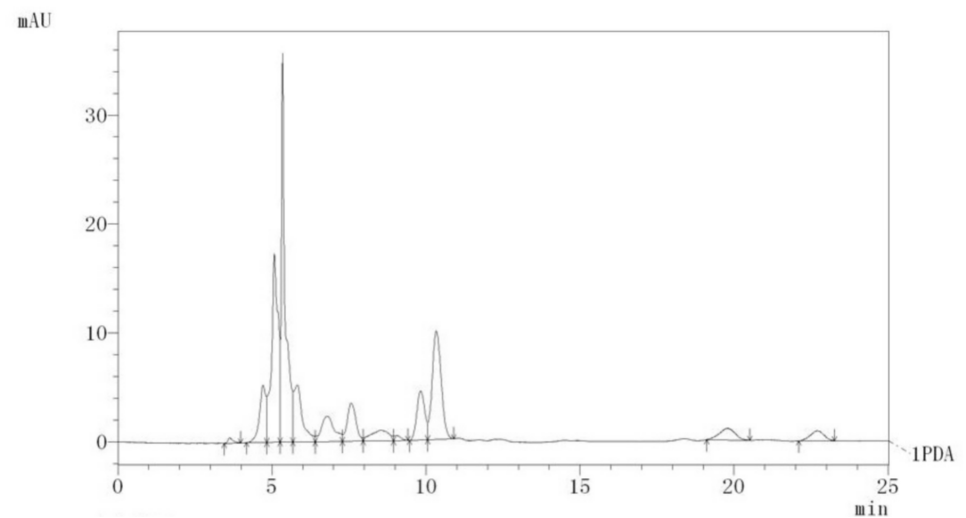


Figure 2. Blood sample after Gd-A injection (PDA / 226nm 4nm).


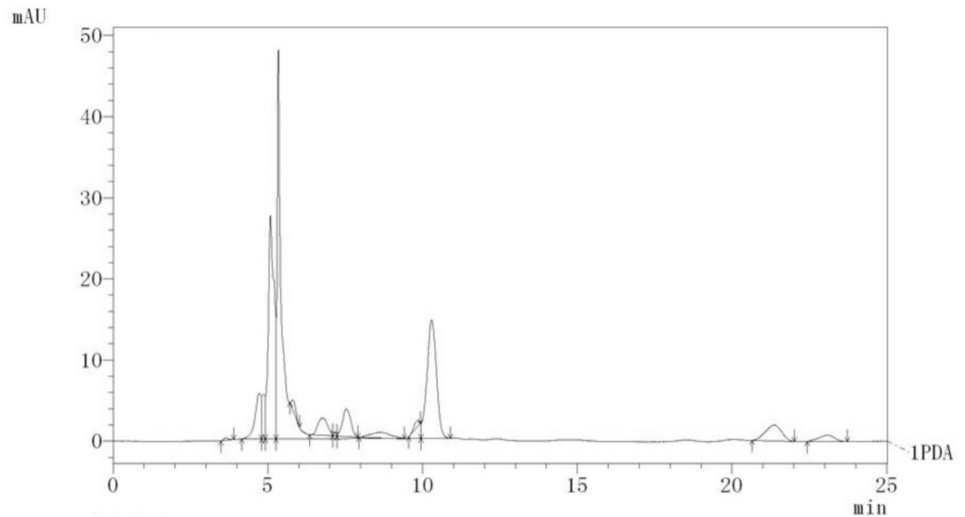


Figure 3. Blood sample after Gd-B injection (PDA / 226nm 4nm).
